# Transcriptome and Cellular Evidence of Depot-Specific Function in Beef Cattle Intramuscular, Subcutaneous, and Visceral Adipose Tissues

**DOI:** 10.3390/biology14070848

**Published:** 2025-07-11

**Authors:** Alexandra P. Tegeler, Hunter R. Ford, Jean Franco Fiallo-Diez, Tainara C. Michelotti, Bradley J. Johnson, Oscar J. Benitez, Dale R. Woerner, Clarissa Strieder-Barboza

**Affiliations:** 1 Department of Veterinary Sciences, Texas Tech University, Lubbock, TX 79409, USA; ategeler@ttu.edu (A.P.T.); hunter.ford@ttu.edu (H.R.F.); jfiallod@ttu.edu (J.F.F.-D.); tainara.michelotti@inrae.fr (T.C.M.); obenitez@ttu.edu (O.J.B.); 2 INRAE, Université Clermont Auvergne, VetAgro Sup, UMR Herbivores, 63122 Saint-Genès-Champanelle, France; 3 Department of Animal and Food Sciences, Texas Tech University, Lubbock, TX 79409, USA; bradley.johnson@ttu.edu (B.J.J.); dale.woerner@ttu.edu (D.R.W.); 4 School of Veterinary Medicine, Texas Tech University, Amarillo, TX 79106, USA

**Keywords:** adipocyte, beef, fat, transcriptome, intramuscular, marbling, visceral, subcutaneous

## Abstract

Deposition of intramuscular adipose tissue (IMAT) is the primary determinant for beef quality grade in the U.S. Accumulation of subcutaneous (SCAT) and visceral (VIAT) adipose tissue precedes that of IMAT and often leads to excessive adiposity in beef cattle. Approaches to increase marbling while limiting subcutaneous and visceral adiposity are limited. Our objective is to define the depot-specific transcriptome profile and adipocyte function in IMAT, SCAT, and VIAT in beef steers. Transcriptomics revealed the upregulation of adipogenic and lipogenic genes in SCAT and VIAT vs. IMAT. Functional transcriptome analysis demonstrated the activation of pathways for lipid metabolic processes and biosynthesis in SCAT, accompanied by increased preadipocyte proliferation, adipocyte size, and insulin responses of SCAT in vitro. While IMAT had a greater abundance of preadipocytes, they proliferated at a lower rate and differentiated into adipocytes that were smaller and less responsive to insulin compared to SCAT. The upregulation of extracellular matrix genes in IMAT suggests that fat accumulation may be limited by the muscle microenvironment. The activation of inflammatory and immune response pathways, combined with a higher abundance of immune cells, highlighted VIAT as an immune-responsive depot. Our findings reveal transcriptional and cellular profiles underlying fat deposition in SCAT, VIAT, and IMAT in beef cattle.

## Simple Summary

Fat inside muscle, known as marbling, is a major factor in determining beef quality. However, before this type of fat develops, cattle often accumulate large amounts of fat under the skin and around internal organs. This can lead to animals being overly fat, which is costly and undesirable. In this study, we compare how fat forms and functions in three areas of the body: inside muscle, under the skin, and around internal organs. We found that fat under the skin and around the organs is more active in producing fat and responding to hormones like insulin. In contrast, muscle fat developed more slowly and had smaller fat cells that were less sensitive to insulin. We also discovered that the area around muscles may naturally limit fat growth due to how its surrounding structure is built. Fat around the internal organs showed signs of more immune activity, suggesting it plays a role in inflammation. These findings provide valuable insight into how different types of fat form in cattle with potential implications for improving meat quality and animal efficiency.

## 1. Introduction

Adipose tissue is an energy storage and endocrine tissue with immunomodulatory functions. Adipogenesis occurs via adipocyte hyperplasia and hypertrophy throughout the life of cattle. Hyperplasia mainly occurs during the fetal and neonatal stages, while adipocyte hypertrophy is of greater importance in later periods [1,2]. These processes are influenced by genetics, nutrition, and management factors that are interlaced and complex to dissect. Intramuscular adipose tissue (IMAT), also known as marbling, refers to the fat deposited between skeletal muscle fibers in cattle [3]. Marbling is a primary factor influencing beef quality grading in the United States [4], increasing the quality of the carcass and consumer acceptability [5]. The linear association between IMAT content and subcutaneous (SCAT) and visceral adipose tissue (VIAT) weights leads to a considerable deposition of undesirable excess fat with every percent of marbling, leading to economic losses to the beef industry [6,7]. For instance, in German Angus cattle, there is an 8 kg increase in SCAT for every 1% increase in the *longissimus* muscle fat content [8]. As economically important as marbling is, the cost of excessive accumulation of subcutaneous backfat and visceral fat in the kidney knob, pelvic area, and heart region (KPH%) in relation to the carcass weight, significantly influences beef yield grade. The estimated loss between Choice-1 and Choice-4 yield grade in a 400-kg carcass is more than USD 100 per head [9]. Understanding the transcriptional and cellular mechanisms that govern lipid accumulation in anatomically distinct adipose depots is essential for strategically modulating fat deposition in beef cattle. This knowledge has direct implications for optimizing carcass composition [10] and enhancing profitability within the beef industry. In particular, Angus and Angus-cross cattle—among the most economically significant breeds in North America—have been selectively bred for superior carcass quality and balanced fat deposition [11], making them an ideal model for such investigations.

Previous adipose tissue RNA sequencing work suggested that the reduced ability of beef cattle to accumulate IMAT compared to SCAT and VIAT is associated with genes encoding increased glucose utilization [12] and the downregulation of adipogenesis regulators, such as *ACACA*, *FASN*, and *ELOVL6* in IMAT [13]. In cattle, stromal vascular cells isolated from IMAT differentiate more slowly and form fewer adipocytes compared to those from subcutaneous and perirenal depots, revealing intrinsic restrictions in IMAT development [14]. In contrast, the pro-adipogenic gene profile of SCAT translates into an increased ability to uptake free fatty acids and expand at higher rates than IMAT, which is contained by muscle fibers [15,16,17]. At the single-cell level, a recent study demonstrated the presence of numerous fibro-adipogenic progenitor cell types in skeletal muscle of beef cattle, and report that decreased marbling associates with a pro-fibrogenic programming of these cells [18]. Notably, pro-adipogenic programming of adipocyte progenitors is driven by *CFD* expression, which was predictive of marbling content [18]. Moreover, cross-species investigations highlight the cellular heterogeneity of adipose depots and demonstrate distinct gene expression signatures and functional properties across depots, including SCAT, IMAT, and VIAT [19]. Overall, these findings demonstrate transcriptional mechanisms underlying adipose tissue deposition in different anatomical locations in beef cattle.

Despite the critical role of marbling in determining beef quality, our understanding of the cellular and molecular mechanisms that regulate adipogenesis in intramuscular versus other fat depots remains limited. While previous studies have highlighted differences in lipid metabolism and gene expression among adipose depots, few have paired transcriptomic profiles with functional cellular assays in cattle. Given the rising interest in precision livestock farming and the application of omics technologies in animal science, comprehensive studies that integrate transcriptomic and cellular assessments across adipose depots to uncover the biology of tissue plasticity and metabolic profiling are required. Our study contributes to addressing this gap by providing novel insights into depot-specific mechanisms of lipid accumulation in SCAT, VIAT, and IMAT of finished Angus-crossed steers, which may inform future breeding and feeding strategies to enhance intramuscular fat deposition. Our study aims to identify the depot-specific transcriptome profile of IMAT, SCAT, and VIAT and define the depot-specific adipocyte metabolic function in vitro in beef steers.

## 2. Materials and Methods

### 2.1. Adipose Tissue Collection and Study Design

**Figure 1:**
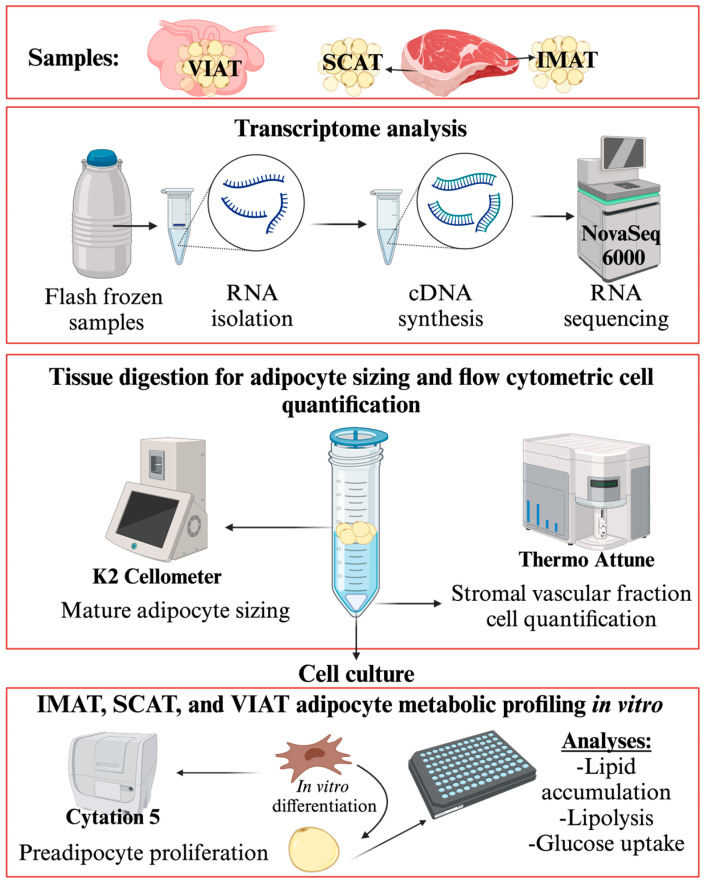
Graphical representation of performed analyses. Created with BioRender.com (License number: OP279HHNB1).

A total of nine crossbred Angus steers were randomly selected during harvest at the Texas Tech University Meat Laboratory. The average live weight of steers was 385 ± 11 kg, and carcasses were graded Choice following the USDA’s official grade standards. While the exact chronological age of the animals was not known, all steers were classified as A-maturity as per the USDA classification. Tissue samples were collected immediately after exsanguination and hide removal. All animals were appraised following USDA standards before slaughter, and there was no sign of distress or illness at the time of entering the facility. A section of the *Longissimus dorsi* (LD) muscle (±500 g) from the 9–11th rib section was excised, placed onto a stainless-steel tray, and immediately placed on ice for performing the manual dissection of IMAT and SCAT (backfat) samples using tweezers and a scalpel. While IMAT dissection was carefully performed, we anticipated contamination of IMAT with muscle tissue, given the location of IMAT between the perimysial space between muscle fibers. Therefore, samples were enriched for IMAT, but not free of muscle tissue. Visceral adipose tissue samples were collected at evisceration from the greater omentum near the larger curvature of the abomasum. Collected samples were placed into Krebs Ringer Buffer (KRB) [1 L DDH_2_O, 7.88 g of NaCL, 0.070 g KH_2_HPO_4_, 0.246 g MgSO_4_·7H_2_O, 0.373 g KCl, 0.991 g glucose, 10 mL Antibiotic/antimycotic (Corning, Cat. No. 30-004CL), 20 mL 1 M HEPES] with gentamycin (50 ug/mL) and rinsed twice before stromal vascular fraction (SVF) extraction. Tissue subsamples of approximately 1 g each were flash frozen in liquid nitrogen for subsequent qRT-PCR and RNA sequencing analysis. A summary of tissue collection and sample analysis workflow is depicted in Figure 1. A sub-sample of four animals was randomly selected for RNA sequencing analysis of IMAT, SCAT, and VIAT samples. While we obtained samples from nine animals, some tissue samples (1) developed contamination during culture and were eliminated from our in vitro assays, or (2) were not large enough to complete all described assays. Thus, some analyses included samples from fewer than 9 animals. The sample size for each assay is described in the caption of the figures.

### 2.2. Adipose Tissue Digestion

Adipose tissue stromal vascular fraction (SVF) was isolated as previously reported by our laboratory [20]. Briefly, approximately 5 g of each adipose tissue sample was minced using 2 mg/mL type II collagenase (Gibco, Waltham, MA, USA, Cat. No. 17101-015) in KRB supplemented with 4% bovine serum albumin (BSA; Fisher Scientific, Waltham, MA, USA, Cat. No. BP1600-100) and placed on an orbital shaker at 37 °C (490 RPM) for 1 h. Samples were then filtered through a pre-wetted 400 µm mesh, and a sample from the upper layer composed of buoyant mature adipocytes was collected for adipocyte size analysis. Next, samples were subsequently filtered through pre-wetted 100 µm and 40 µm strainers (Fisher Scientific, Waltham, MA, USA, Cat. No. 22-363-549 and 22-363-547) and spun down at 800 rcf (20 °C for 5 min). The resulting pellet was resuspended in 1× erythrocyte lysis buffer (Biolegend, San Diego, CA, USA, Cat. No. 420301) for 6 min at room temperature. After incubation, cold 1× PBS was added to stop the lysis, and samples were centrifuged at 800 rcf (20 °C for 5 min). The cell pellet was resuspended in 2 mL of preadipocyte growth medium, composed of Dulbecco’s Modified Eagle’s Medium (DMEM/F12 50:50; Corning, Corning, NY, USA, Cat No. MT10090CV) supplemented with 10% FBS (Corning, Waltham, MA, USA, Cat. No. 35-016-CV), 1% Antibiotic-Antimycotic (Gibco, Waltham, MA, USA, Catalog No. 15240112), 100 µM Ascorbic Acid (Sigma-Aldrich, St. Louis, MO, USA, Cat. No. A4544-25G), 33 μm/L of biotin (Sigma-Aldrich, St. Louis, MO, USA, Cat. No. B4639-500 mg), and 20 mmol/L of HEPES 10M (pH = 7.3, Thermo Scientific, Waltham, MA, USA, Cat. No. J16924.K2). Cell concentration and viability were analyzed in an automated counter (Countess 3, Life Technologies Inc., Carlsbad, CA, USA). A portion of the SVF sample was collected for flow cytometry analysis and resuspended at 1 × 10^6^ cells/mL in FACS buffer [1× PBS with 0.1% sodium azide (NaN3; Sigma-Aldrich, Cat. No. S2002-25G) and 2% FBS (Gibco, Waltham, MA, USA, Cat. No. 16140071)]. Remaining cells were placed into a T-25 flask with preadipocyte growth medium and placed into a 37 °C incubator in a humidified atmosphere with 5% CO_2_, with media replacement every 48 h. Preadipocytes were obtained by outgrowth of plastic-adherent cells from the SVF cells after two serial passages in culture flasks. 

### 2.3. Adipocyte Culture

Adipocyte culture was performed as previously described by our laboratory [21]. Briefly, confluent preadipocytes were detached from the cell culture flasks using 0.05% trypsin (Thermofisher Scientific, Waltham, MA, USA, Cat. No. 25300054) seeded into cell culture-treated plates at 5 × 10^4^ cells/well in 24-well plates and 2 × 10^4^ cells/well in 96-well plates containing a basement membrane (Thermofisher Scientific, Waltham, MA, USA, Cat. No. A1569601) and grown to confluency over 2 days in preadipocyte growth medium. Adipogenic induction was performed using preadipocyte growth medium supplemented with 5 μmol/L of troglitazone (AdipoGen Life Sciences, San Diego, CA, USA, Cat. No. AG-CR1-3565-M005), 0.5 mmol/L of 2 isobutyl-1-methylaxanthine (IBMX; AdipoGen Life Sciences, San Diego, CA, USA, Cat. No. AG-CR1-3512-G001), and the following reagents from Sigma-Aldrich, St. Louis, MO, USA: 0.5 μg/mL of insulin (Cat. No. 10516-5 mL), 10 mM acetate (Cat. No. 3863-50 mL), and 1 μmol/L of dexamethasone (Cat. No. D2915-100 mg). After the first 48 h, IBMX and dexamethasone were removed from the medium, and cells were allowed to differentiate for an additional 12 days (14 days total). Medium replacement was performed every 48 h.

### 2.4. RNA Isolation

RNA was extracted from IMAT, SCAT, and VIAT samples using Qiagen Lipid Minikit (Qiagen, Hilden, Germany, Cat. No. 74804). In short, 50–75 mg of frozen adipose tissue was placed into a polystyrene tube that contained 2 zirconium beads (Benchmark, Tempe, AZ, USA, Cat. No. D1132-30TP). Then, 1 mL of Trizol reagent (Invitrogen, Waltham, MA, USA, Cat. No. 10296028) was added to each tube right before placing it onto the bead blaster (Benchmark Scientific Inc., Sayreville, NJ, USA). The tissue was then pulverized at 400 m/s in 30-s intervals, and 200 µL of chloroform (Invitrogen, Waltham, MA, USA, Cat. No. AM9732) was added. Samples were centrifuged, and the clear aqueous phase was collected and placed into a spin column, washed with the supplied buffers, and RNA was rinsed out of the column using RNase-free water. All RNA samples were isolated in our laboratory at Texas Tech University and screened for RNA concentration and 260/280 ratio for purity via a Take3 plate in a Cytation 5 (Agilent). The RNA integrity number (RIN) was assessed using the RNA Nano 6000 Assay Kit of the Bioanalyzer 2100 system (Agilent Technologies, Santa Clara, CA, USA) and reported in Supplementary Table S1.

### 2.5. RNA Sequencing

First-strand cDNA was synthesized from IMAT, SCAT, and VIAT RNA samples using random hexamer primer and M-MuLV Reverse Transcriptase (RNase H-), using RNA samples as described above. Second-strand cDNA synthesis was subsequently performed using DNA Polymerase I and RNase H. PCR was performed with Phusion High-Fidelity DNA polymerase, Universal PCR primers, and Index (X) Primer. PCR products were purified (AMPure XP system), and library quality was assessed on the Agilent Bioanalyzer 2100 system. The clustering of the index-coded samples was performed on a cBot Cluster Generation System using TruSeq PE Cluster Kit v3-cBot-HS (Illumina, Inc., San Diego, CA, USA) according to the manufacturer’s instructions. After cluster generation, the library preparations were sequenced on an Illumina Novaseq 6000 S4 platform (Illumina, Inc., San Diego, CA, USA) to generate 30 million stranded paired-end reads (2 × 150 nt) per sample. Quality control was performed by removing (1) reads containing adapters, (2) reads when uncertain nucleotides constitute more than 10% of either read (N > 10%), and (3) low-quality nucleotides (Base Quality less than (4) constitute more than 50 per cent of the read). Clean reads were mapped to the *Bos taurus* genome ARS-UCD1.3 using HISAT2 and annotated to NCBI RefSeq assembly accession: GCF_002263795.2. Gene expression analysis was performed using the DESeq2 package in R (version 4.3.2). Differentially expressed genes (DEGs) were denoted as genes with |log_2_(FoldChange)| ≥ 0.5 and padj < 0.05. Cluster analysis was used to find genes with similar expression patterns in the different AT depots. Functional analysis of enriched KEGG pathways and Gene Ontology (GO) Biological Process (BP) terms was performed using the clusterProfiler (version 3.2) package in R using the whole IMAT, SCAT, and VIAT transcriptome.

### 2.6. Quantitative Real Time PCR

Targeted gene expression analysis in IMAT, SCAT, and VIAT samples was performed by quantitative real-time PCR (qPCR). Selected genes for qPCR analysis were based on key markers of adipogenesis and lipogenesis (*ADIPOQ*, *LEP*, *DGAT1*, *LIPE*, *FASN*, and *PPARG*), inflammation (*TNF*, *IL6*, *TGFB1*), and ECM markers (*FN1*, *TIMP2*, *LOX*). cDNA was synthesized using the High-Capacity cDNA kit (Applied Biosystems, Waltham, MA, USA, Cat. No. 4374967) in a MiniAmpPlus thermal cycler (Applied Biosystems, Cat. No. A37835). The qPCR was conducted with hydrolysis probes (TaqMan^®^; Table 1; Life Technologies Inc., Carlsbad, CA, USA) in a QuantStudio 6 Pro (Applied Biosystems, Waltham, MA, USA, Cat. No. A43180). The qPCR reactions were prepared in 384-well plates (Catalog No. 4483285, Thermofisher Scientific, Waltham, MA, USA) at 10 µL/well containing 5 µL of TaqMan Universal Master Mix (Applied Biosystems, Waltham, MA, USA, Cat. No. 4304437), 0.5 µL of gene expression TaqMan assay, 2 uL of sample cDNA (based on 5 ng of initial RNA), and 2.5 µL of Rnase-free water. We evaluated the stability of 4 reference genes via one-way ANOVA, including *EIF3K*, *RPS9*, *B2M*, and *ACTB*. Only *EIF3K* and *RPS9* were stable among depots and used for normalization of target genes. The *Cq* (quantification cycle) values of the target genes were converted to normalized relative gene expression using the qBase relative quantification framework, as established by Hellemans et al. [22].

### 2.7. Adipogenic Function

Depot-specific adipogenic function was evaluated by the (1) abundance of preadipocytes via flow cytometry, (2) preadipocyte proliferation, (3) adipocyte size, and (4) adipocyte lipid accumulation, as follows.

#### 2.7.1. Adipose Tissue Cellular Composition by Flow Cytometry

Flow cytometry was performed to define the abundance of preadipocytes (CD31^−^CD45^−^), endothelial cells (CD31^+^CD45^−^), and immune cells (CD31^−^CD45^+^), as reported in our previous study [23]. Briefly, 1 million cells were suspended in FACS medium in 5 mL polystyrene tubes and centrifuged at 300× *g* at 4 °C for 5 min. Cells were stained with 1.5 µg of FITC anti-bovine CD31 (ThermoScientific, Waltham, MA, USA, Cat. No. MA1-80360) and 10 µL of PE anti-bovine CD45 (ThermoScientific, Waltham, MA, USA, Cat. No. MA1-81458) at 4 °C for 30 min. Samples were then washed with FACS buffer and centrifuged 3 times, with the supernatant removed after each centrifugation. Next, stained cells were fixed using cold 4% paraformaldehyde (PFA) and placed at 4 °C protected from light for a maximum of 4 days before performing flow cytometry analysis in an Attune NxT Flow Cytometer (Thermo Fisher Scientific, Waltham, MA, USA). When ready to perform flow cytometry, 250 µL of FACS buffer was added to the tubes to increase the volume to approximately 500 µL. Unstained cells and fluorescence minus one control were used as controls. Data are presented as a percentage of cells out of the total cell number in the adipose tissue sample stromal vascular fraction.

#### 2.7.2. Preadipocyte Proliferation Assay

Proliferation over time in vitro was used as a measurement of preadipocyte hyperplasia, a key adipogenic function, as described by our laboratory [20]. Briefly, preadipocytes were plated into 24-well plates at a seeding density of 1 × 10^4^ cells/well in duplicates and allowed to attach for 24 h. Cell proliferation was determined by automated confluency estimation and cell counts using a Cytation 5 multi-mode reader (Biotek, Santa Clara, CA, USA). One brightfield image per quadrant of the well was obtained using a 4× objective at the same well location every 48 h for 7 days. Images were processed to reduce background noise and increase contrast, and confluence was measured using Gen5 Image Prime v.3.08 (Agilent) software.

#### 2.7.3. Mature Adipocyte Sizing

The measurement of adipocyte area was used to assess the cells’ ability to accumulate lipids, as an assessment of cell hypertrophy. Sizing was performed in buoyant adipocytes collected from the upper layer during AT digestion with collagenase. Briefly, 200 µL of the sample was transferred to a 1 mL Eppendorf tube, mixed with 1:1 1× PBS, counted and sized using a K2 Cellometer (Nexcelom, Lawrence, MA, USA).

#### 2.7.4. Adipocyte Lipid Accumulation

To qualitatively assess adipocyte lipid accumulation, we stained in vitro differentiated adipocytes with HCS LipidTOX Red Neutral Lipid Stain (Thermoscientific, Waltham, MA, USA, Cat. No. H34476) [20]. Briefly, preadipocytes were seeded into 96-well plates at a density of 3 × 10^4^/well and induced to differentiate for 14 days. Adipocytes were washed gently three times with 1% PBS and fixed using PFA for 30 min protected from light at room temperature. After fixing, the cells were washed with 1% PBS three times, and 100 µL of 1:200 HCS LipidTOX stain was added to each well for 30 min in a dark room. Wells were washed, and 100 µL of 1 µg/mL of diamidino-2-phenylindole (DAPI) was added for 15 min. Images of stained lipid droplets were obtained using Cytation 5 (Agilent) at 20× magnification.

#### 2.7.5. Adipocyte Insulin Response

To evaluate adipocyte insulin responses, an insulin-stimulated glucose uptake assay was performed using a 2-NBDG Glucose Uptake Assay Kit (Cayman, Ann Arbor, MI, USA, Cat. No. 600470), as described by our laboratory [20]. Briefly, preadipocytes were seeded at a density of 3 × 10^4^ cells per well in black walled, 96-well plate with a clear bottom and induced to differentiate as described above. On the 13th day of differentiation, cells were serum- and glucose-starved for 16 h. Adipocytes were then treated with 1 µg/mL insulin in PBS and incubated for 20 min at 37 °C. Non-treated cells served as a control. Next, 200 µg/mL of 2-NBDG was added to each well for 20 min and incubated at 37 °C. Plates were spun for 5 min at 400× *g* at room temperature, supernatant was removed, cell-based assay buffer was added, and the plate was spun again for 5 min at 400× *g* at room temperature. After centrifugation, cell-based assay buffer was added to the wells, and fluorescence was measured in a Cytation 5 (Agilent, Santa Clara, CA, USA) multi-mode plate reader. Measured relative fluorescent units (RFU) per well were calibrated by the number of cells as assessed by imaging analysis of individual wells using Cytation 5 (Agilent, Santa Clara, CA, USA).

### 2.8. Statistical Analyses

For adipocyte sizing, the frequency of distribution of adipocyte area was calculated and stratified into 60 μm intervals (from <60 μm^2^, 60–120 μm^2^, and >120 μm^2^) [20]. The effect of AT depot on adipocyte size frequency was assessed with the PROC FREQ procedure in SAS (version 9.4; SAS Institute Inc., Cary, NC, USA) using the chi-square test with significance declared at a *p* value ≤0.05 and tendency at a *p* value <0.10. The frequency of adipocytes in each of our three size classifications (<60 μm^2^, 60–120 μm^2^, and >120 μm^2^) was also calculated for each sample and compared between depots using one-way ANOVA and Tukey multiple comparison analysis in GraphPad Prism 10. Statistical analyses of qRT-PCR, glucose uptake, and flow cytometry were performed in GraphPad Prism 10 using one-way ANOVA and Tukey multiple comparison analysis. Adipocyte proliferation was analyzed in GraphPad Prism 10 using an RM two-way ANOVA with the Geisser–Greenhouse correction, followed by multiple comparisons analysis comparing depots throughout time. Normality was checked, and if *p*-values <0.05 were detected, data were ln-transformed and non-parametric tests were performed, such as the Kruskal–Wallis test. Outliers were detected and removed using the ROUT (Robust Regression and Outlier removal) method with a stringency coefficient of Q = 1% in GraphPad Prism. This method combines nonlinear regression with false discovery rate (FDR) control to identify data points that deviate significantly from the overall distribution while minimizing the risk of false positives. We selected a stringent threshold (Q = 1%) to ensure that only data points with a high likelihood of being true outliers were excluded, thus preserving the integrity of the biological variability. This is particularly important in exploratory studies with small sample sizes, as was the case here, where retaining authentic biological signals while mitigating undue statistical influence from extreme values is critical. *p*-values ≤ 0.05 were considered significant, while *p*-values ≥ 0.051 and ≤0.10 were identified as tendencies. Significance was declared at a *p*-value ≤0.05 and tendencies at a *p*-value <0.10.

## 3. Results

### 3.1. Transcriptome Analysis Reveals Differences in Key Genes Regulating Adipocyte Function, Extracellular Matrix Deposition, and Inflammation Among IMAT, SCAT, and VIAT

We performed bulk RNA sequencing analysis to identify depot-specific transcriptomic differences between IMAT, SCAT, and VIAT of beef steers. Data analysis revealed that one VIAT sample (Beef 6) differed from the other VIAT samples as revealed by a principal component analysis (PCA; Supplementary Figure S1). RIN (6.8) and quality of sequencing results revealed no issues with the integrity of this sample. Similarly, antemortem and postmortem inspection of this animal following USDA standards did not indicate any apparent health or carcass issues, respectively. Additionally, analysis of the top up- and downregulated genes with and without the inclusion of this sample was largely similar (Supplementary Table S2). Thus, we opted to include this sample in the data analysis.

**Figure 2:**
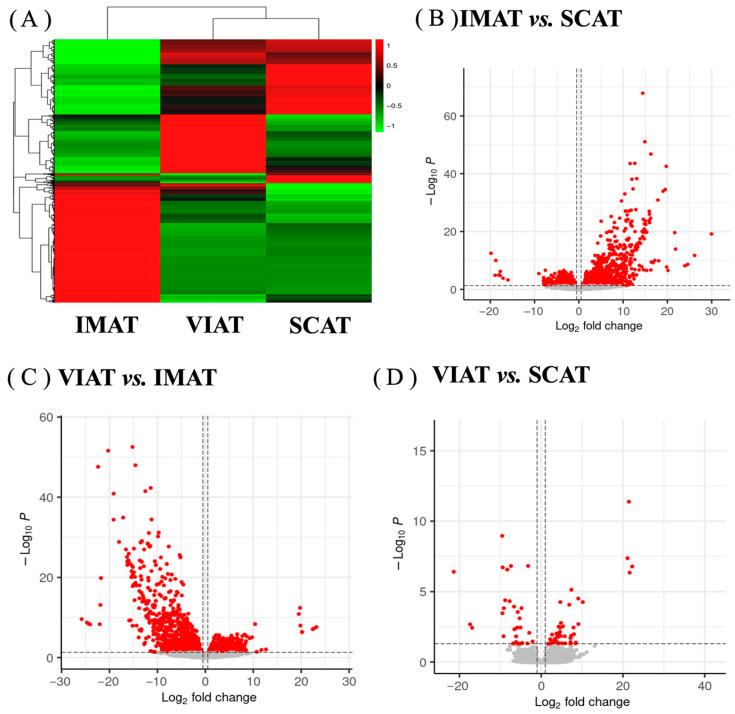
Gene clustering in intramuscular (IMAT), subcutaneous (SCAT), and visceral (VIAT) adipose tissues of beef cattle (*n* = 4). (**A**) Whole transcriptome heatmap of IMAT, VIAT, and SCAT gene expression. (**B**–**D**) Volcano plots comparisons of differentially expressed genes in IMAT vs. SCAT, VIAT vs. IMAT, and VIAT vs. SCAT. Red dots with positive fold changes on the ‘x’ axis refer to upregulated DEGs, while red dots with negative fold changes refer to downregulated DEGs.

Heat map clustering of genes revealed similarities in the overall transcriptome profiles of SCAT and VIAT, with greater differences between both depots and IMAT (Figure 2A). Depot-specific gene profiles yielded 1761 DEGs between IMAT and SCAT (1176 upregulated and 585 downregulated; Figure 2B; Supplementary Table S3), 1972 DEGs between VIAT and IMAT (791 upregulated and 1181 downregulated; Figure 2C; Supplementary Table S3), and only 81 DEGs (50 upregulated and 31 downregulated; Figure 2D; Supplementary Table S3) between VIAT and SCAT, thus demonstrating remarkable differences in the transcriptome profile between IMAT and VIAT/SCAT.

The top 10 most significant DEGs (filtered by combined higher log_2_fold change and lowest adjusted *p*-value) between IMAT vs. SCAT, VIAT vs. IMAT, and VIAT vs. SCAT are reported in Table 2, Table 3 and Table 4, respectively. In comparison to both SCAT and VIAT, the most significant DEGs in IMAT included muscle-specific proteins and muscle contraction-related proteins, such as *ACTA1*, *CKM*, *MB*, and *TNNC2* (Table 2 and Table 3), which reflects the interlaced proximity of IMAT and skeletal muscle in cattle. The DEG with the highest fold change increase in IMAT vs. SCAT was *UCP3* (29.95 log_2_ fold change), while *SCRT1* (−19.85 log_2_ fold change), a regulator of insulin expression, was the most downregulated gene in IMAT compared with both SCAT (−19.85 log_2_ fold change) and VIAT (−19.76 log_2_ fold change; Table 2 and Table 3; Supplementary Table S3).

Classical markers of adipocyte function, such as *LEP*, *ADIPOQ*, *PLIN1*, *PLIN4*, *LPIN2*, *CD36*, and *DGAT2*, were downregulated in IMAT vs. SCAT, accompanied by the downregulation of the adipogenesis regulators *CEBPA* and *PPARG-TSEN2* (Supplementary Table S3). Similarly, in IMAT vs. VIAT comparison, we observed the downregulation of *LEP*, *PLIN1*, *LIPE*, *LPIN2*, *ACACA*, and *DGAT2*, in addition to *CFD* (complement factor D/adipsin), an adipokine and marker of progenitor cells in bovine muscle [18] and human adipose tissue [24] (Supplementary Table S3). These findings suggested a decreased adipogenic/lipogenic function in IMAT vs. SCAT and VIAT.

Our transcriptome data also suggested decreased insulin sensitivity of IMAT compared to SCAT and VIAT. Specifically, compared to SCAT, IMAT showed a downregulation of *SCRT1*, *TRARG1* (trafficking regulator of GLUT4-1), a regulator of glucose uptake, and *ADIPOQ*, a recognized endogenous insulin-sensitizing adipokine [25]. In IMAT vs. VIAT, in addition to the downregulation of *SCRT1* and *TRARG1*, *INSRR* (insulin receptor-related receptor), which can stimulate glucose uptake in adipocytes [26], was also decreased (Supplementary Table S3).

Notably, we observed differences in genes associated with the extracellular matrix (ECM) deposition and remodeling. For instance, in IMAT vs. SCAT, there was a decrease in the expression of *MMP14*, a metalloproteinase involved in the degradation of the extracellular matrix (ECM), and *TIMP2*, which inhibits MMPs. Additionally, there was an upregulation of collagens (e.g., *COL11A2*, *COL2A1*, *COL5A3*) and several integrins (e.g., *ITGB6*, *ITGA8*) that mediate the interactions between cells and the ECM. Similarly, in IMAT vs. VIAT, we observed decreased *MMP14* and *TIMP4* and increased collagens (e.g., *COL13A1*, *COL2A1*). Notably, the connective tissue growth factor (*CTGF*), linked to increased ECM deposition and insulin resistance in humans [27], was upregulated in IMAT vs. VIAT (Supplementary Table S3).

Our transcriptome analysis also revealed an increased expression of genes associated with vascular function in SCAT and VIAT vs. IMAT compared to both, including *ADTRP*, *KDR*, and *THBS2* (Supplementary Table S3). There was an upregulation of *ICAM1* in SCAT and VIAT vs. IMAT, which facilitates the adhesion of leukocytes to endothelial cells as part of an inflammatory response. In accordance, comparison of VIAT vs. IMAT transcriptome revealed the upregulation of numerous DEGs with pro-inflammatory roles, including complement proteins (*C3*, *C7*), chemokines (*CCR8*, *CCL5*), and ceruloplasmin (Supplementary Table S3) among the top 50 most significant upregulated genes. Other known markers of adaptive immune responses, such as *IRF4*, *CD84*, *CD52*, *CD53*, as well as *TNF*, a key inflammatory activator, were also among the top 150 DEGs upregulated in VIAT vs. IMAT (Supplementary Table S3). The pro-inflammatory transcriptional profile of VIAT was less evident when compared to SCAT. However, DEG analysis revealed the increased expression of *CFI*, *IFITM10*, *TNFRSF9*, and *TNFRSF19* between VIAT and SCAT.

### 3.2. Enrichment Analysis Highlights Pathways of Lipid Metabolism and Immune Responses Differentiating IMAT, SCAT, and VIAT

**Figure 3:**
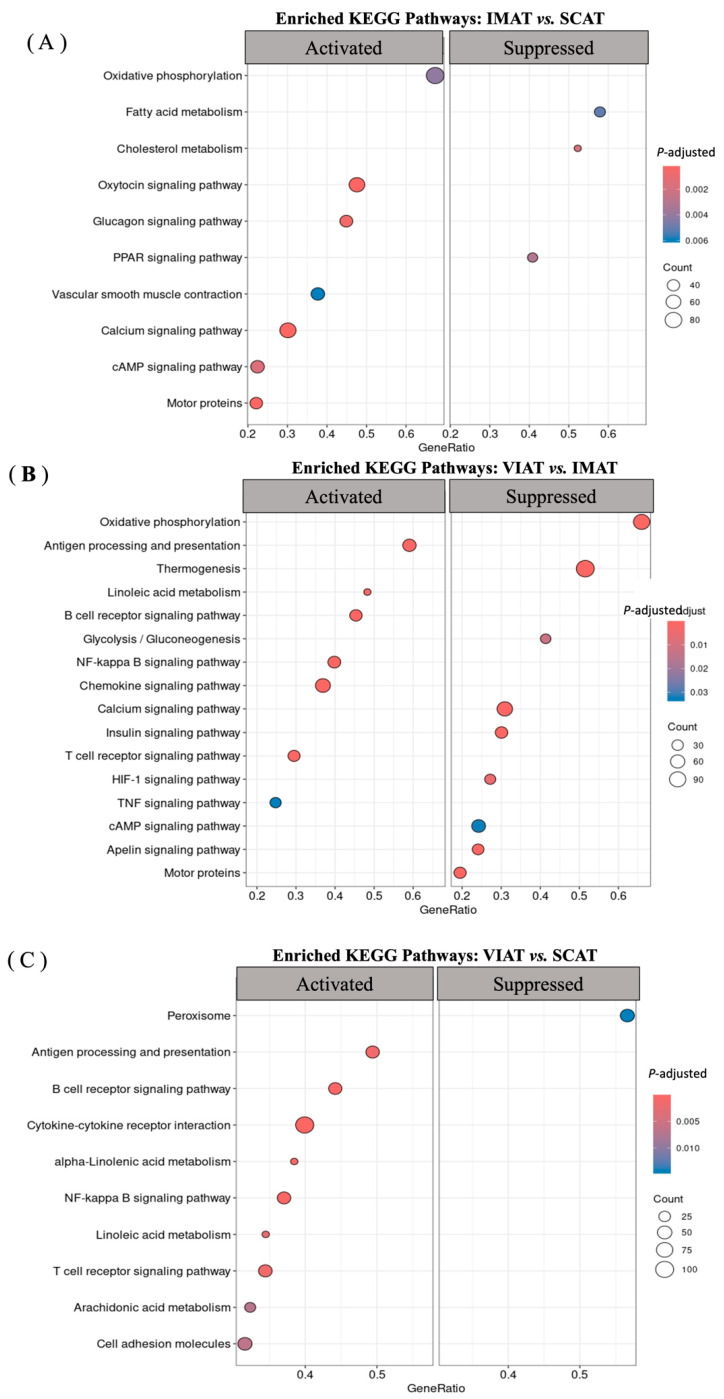
KEGG pathway analysis from intramuscular (IMAT), subcutaneous (SCAT), and visceral (VIAT) adipose tissues RNA sequencing transcriptome (*n* = 4) from beef steers. Activated and suppressed KEGG pathways in (**A**) IMAT vs. SCAT, (**B**) VIAT vs. IMAT, and (**C**) VIAT vs. SCAT.

Enrichment analyses were performed with IMAT, SCAT, and VIAT whole transcriptome via the KEGG pathway (Figure 3A–C; Supplementary Table S4) and GO terms analyses (Supplementary Table S5). Examination of the top 20 enriched KEGG pathways comparing IMAT to SCAT (Figure 3A) ratified DEG analysis and suggested that lipid accumulation in IMAT adipocytes may be limited by the muscle microenvironment. This was implied by the suppression of fatty acid and cholesterol metabolism and PPAR signaling pathways, mediated by genes such as *FASN*, *SCD*, *PLIN4*, and *ADIPOQ*, which are key regulators of adipogenesis and lipogenesis downregulated in IMAT vs. SCAT (Supplementary Table S3). Accordingly, GO biological processes (Supplementary Table S5) downregulated in IMAT vs. SCAT included many lipid metabolic processes associated with fatty acid biosynthesis and diacylglycerol metabolism, associated with the enrichment of downregulated DEGs such as *DGAT1*, *LIPE*, *INSIG1*, *C3*, and *LEP*. In contrast, activated KEGG pathways (Figure 3A) in IMAT vs. SCAT included motor proteins, calcium signaling, and oxidative phosphorylation, enriched for mitochondrial genes (Supplementary Table S4), while activated GO terms were associated with muscle development and function (Supplementary Table S5), mostly mediated by signature muscle markers, such as *ACTA1*, *TNNC1*, and *MYOZ*. The thermogenesis pathway was among the top 30 most activated KEGG pathways in IMAT vs. SCAT, highlighted by the enriched expression of of *PRDM16*, a marker of thermogenic adipocytes, and several mitochondrial genes (*COX*, *ATP*, and *NDUF*), which were also implicated in the upregulation of the oxidative phosphorylation pathway (Supplementary Table S4). Thermogenesis and oxidative phosphorylation were also among the top suppressed pathways in VIAT vs. IMAT (Figure 3B, Supplementary Table S4). In addition, the insulin and the apelin signaling pathways were also suppressed in VIAT vs. IMAT, enriched for downregulated DEGs in VIAT, such as *CALM* and *PRKACA* (Supplementary Table S3). Similar to the top activated GO terms in IMAT vs. SCAT, the top suppressed GO terms in VIAT vs. IMAT were associated with muscle development and function (Supplementary Table S5). Altogether, these results indicate SCAT and VIAT are primary energy storage depots, while IMAT appears to generate energy through enhanced mitochondrial and thermogenic gene expression, potentially influenced by its location within skeletal muscle.

Functional analysis of the VIAT transcriptome revealed an activation of KEGG pathways and GO terms associated with immune function when compared with either IMAT or SCAT (Figure 3B,C; Supplementary Table S4). Notably, the antigen processing and presentation, B Cell Receptor Signaling, NF-Kappa B Signaling, and T Cell Receptor Signaling Pathways were all activated in VIAT compared to both IMAT (Figure 3B) and SCAT (Figure 3C), containing *TNF*, *CCL4*, and *TNFRSF9* as enriched core genes. The immune profile of VIAT was also consistent with the top enriched GO terms in VIAT compared to IMAT, which included many GO terms associated with the immune response and immune activation (Supplementary Table S5). Interestingly, additional activated GO terms in VIAT vs. SCAT were associated with metabolism and secretion (Supplementary Table S5) and the increased expression of *SFRP1* (Supplementary Table S3), a DEG between VIAT and SCAT, while GO terms related to muscle function and development were suppressed (Supplementary Table S5).

**Figure 4:**
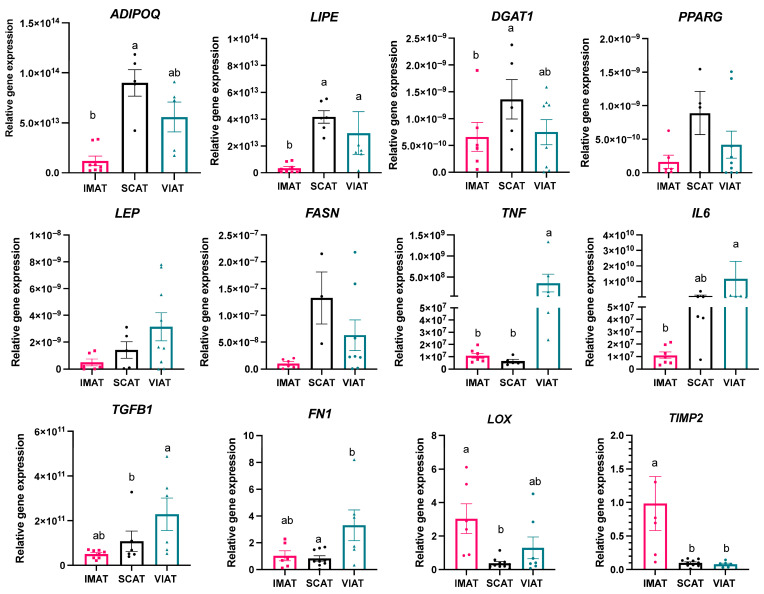
Relative gene expression of targeted adipogenic/lipogenic genes (*ADIPOQ*, *LIPE*, *DGAT1*, *PPARG*, *LEP*, and *FASN*), inflammatory markers (*TNF*, *IL6*, *TGFB1*), and extracellular matrix deposition markers (*FN1*, *LOX*, *TIM2*) in SCAT, IMAT, and VIAT of beef steers (IMAT: *n* = 6–7; SCAT: *n* = 6; VIAT: *n* = 6–7) analyzed by targeted RT-PCR. Different letters denote *p* < 0.05.

To corroborate the pro-adipogenic/lipogenic profile of SCAT, the pro-inflammatory profile of VIAT, and their potential linkage to differences in adipose tissue ECM, we performed RT-qPCR analysis of key gene markers of adipogenesis and lipogenesis, inflammation, and ECM remodeling/deposition (Figure 4). With the exception of *DGAT1*, which was enriched in distinct KEGG pathways and GO terms of lipid metabolism, the expression of *ADIPOQ*, *LIPE*, *LEP*, and *FASN* was detected among the DEG list and, thus, selected for targeted PCR. We observed increased expression of *ADIPOQ* and *DGAT1* in SCAT compared to IMAT (*p* = 0.0001; *p* = 0.0171), but not VIAT, while *LIPE* was increased in SCAT (*p* = 0.0002) and VIAT (*p* = 0.0055) compared to IMAT (Figure 4). We did not detect differences in the expression of *LEP*, *PPARG*, and *FASN* among depots, even though we observed statistical tendencies (*p*-values < 0.20) in *PPARG* and *FASN*, being increased in SCAT vs. IMAT, and *LEP* increased in VIAT. In addition to *TNF*, which was significantly upregulated in VIAT vs. IMAT transcriptome data, we also evaluated the expression of *IL6*, *TGFB1*, and *FN1* as markers of inflammatory response, which were highlighted in distinct KEGG pathways and GO terms (Supplementary Tables S4 and S5). For example, *FN1* expression was enriched in multiple upregulated GO terms between VIAT vs. IMAT (Supplementary Table S5), including inflammatory response, acute inflammatory response, and defense response, and KEGG pathways between VIAT vs. IMAT, and VIAT vs. SCAT (Supplementary Table S4). Expression of the pro-inflammatory markers *TNF* and *FN1* was higher in VIAT compared to both SCAT (*p* = 0.0004; and *p* = 0.0566) and IMAT (*p* = 0.0012; *p* = 0.0479; Figure 4), while the expression of *IL6* was increased in VIAT compared to IMAT (*p* = 0.0020), and *TGFB1* was increased in VIAT vs. SCAT (*p* = 0.0119).

We also evaluated the gene expression of lysyl oxidase (*LOX*), an enzyme that catalyzes the cross-linking of collagen fibers within ECM, and is thus associated with tissue fibrosis [28], and the tissue inhibitor of metalloproteinase-2 (*TIMP2*), high levels of which can inhibit the breakdown of ECM proteins and promote fibrosis development. Notably, we observed higher gene expression of both *LOX* (*p* = 0.0078) and *TIMP2* (*p* = 0.0225) in IMAT vs. SCAT. The expression of *TIMP2* was also increased in IMAT vs. VIAT (*p* = 0.0075), but similar between SCAT and VIAT (*p* > 0.05), while *LOX* did not differ between IMAT and VIAT, nor between SCAT and VIAT (*p* > 0.05). Overall, our targeted RT-PCR results corroborate with the transcriptome results, pointing to a decreased adipogenic/lipogenic function in IMAT vs. SCAT and VIAT, an increased pro-inflammatory response in VIAT, and a potential increase in ECM deposition in IMAT, particularly when compared with SCAT.

### 3.3. Profiling of IMAT, SCAT, and VIAT Adipocytes Imply Depot-Specific Cell Function

**Figure 5:**
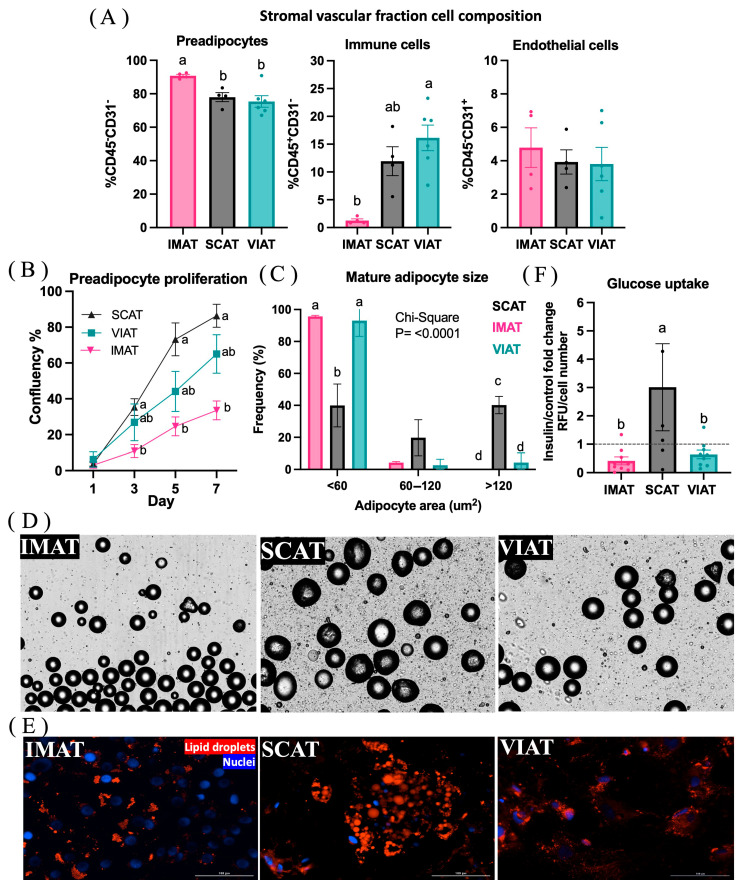
Adipogenic assessment and metabolic phenotype of adipocytes from intramuscular (IMAT), subcutaneous (SCAT), and visceral (VIAT) adipose tissue of beef cattle. (**A**) Abundance of CD31^−^/CD45^−^ (preadipocytes), CD31^−^/CD45^+^ (immune cells), and CD31^+^/CD45^−^ (endothelial cells) in SCAT (*n* = 4), IMAT (*n* = 4), and VIAT (*n* = 6) stromal vascular fraction (SVF) identified by flow cytometry analysis. Percentages were calculated as a proportion of total SVF cells. (**B**) In vitro proliferation of IMAT, SCAT, and VIAT preadipocytes from beef steers (*n* = 6) assessed by automated confluency measurement (Cytation 5, Agilent, Santa Clara, CA, USA). Distinct letters denote a statistical difference among depots on any specific day. (**C**) Mature adipocyte area collected from IMAT, SCAT, and VIAT SVF during collagenase digestion (*n* = 6) and measured by a K2 Cellometer (Nexcelom). (**D**) Representative K2 Cellometer images of mature adipocytes (5× magnification) measured in C. (**E**) Representative images of SCAT, IMAT, and VIAT adipocyte lipid accumulation. Adipocyte lipid droplets were stained with LipidTox (red) and nuclei with DAPI (blue). Images were obtained at 20× magnification with an epi-fluorescent microscope (Cytation 5, Agilent, Santa Clara, CA, USA). Scale bars: 100 microns. (**F**) Insulin stimulated glucose uptake of SCAT (*n* = 5), IMAT (*n* = 9), and VIAT (*n* = 9) adipocytes. Data are shown as relative fluorescence units (RFU) fold change of insulin over control (non-stimulated cells) corrected by cell number. Statistical analysis was performed on log_2_-transformed values. Dashed line indicates fold change = 1 (no change). (**A**,**C**,**F**): Different letters denote statistical difference, whereas the same letter indicates no difference between samples.

To further investigate the differences in adipogenesis/lipogenesis and insulin sensitivity of IMAT, SCAT, and VIAT suggested by the transcriptomic analysis, we performed cell-based assays in vitro (Figure 5). First, flow cytometry analysis of IMAT, SCAT, and VIAT SVF evaluated the abundance of main adipose tissue cell types, including preadipocytes, immune cells, and endothelial cells based on the selective expression of CD45 and CD31 markers (Figure 5A). Despite the decreased expression of adipogenic genes in IMAT compared to VIAT and SCAT demonstrated by our transcriptome and targeted qPCR analyses, there was a higher proportion of preadipocytes (CD31^−^CD45^−^) in IMAT compared to both SCAT (*p* = 0.0398) and VIAT (*p* = 0.0087); however, no differences were observed between SCAT and VIAT (*p* = 0.8135; Figure 5A). In agreement with our RNAseq analysis and targeted qPCR for immune response-associated markers, immune cells (CD31^−^/CD45^+^) were more abundant in VIAT compared to IMAT (*p* = 0.0430), but no differences were detected between VIAT and SCAT (*p* = 0.9972) or between SCAT and IMAT (*p* = 0.4922; Figure 5A). While our transcriptome findings suggest changes in the vascularization between depots, no differences in endothelial cell proportions were detected among IMAT, SCAT, and VIAT (*p* > 0.05).

Second, we assessed depot-specific adipogenic function by evaluating preadipocyte proliferation and adipocyte size paired with lipid accumulation imaging. Despite the greater abundance of preadipocytes in IMAT, the proliferative rate of IMAT cells in vitro was decreased compared to SCAT (*p <* 0.05), but did not differ from VIAT preadipocytes, which was also similar to SCAT (Figure 5B). Moreover, preadipocyte proliferation was similar between VIAT and SCAT depots at all time points (*p* > 0.05). Aligning with the pro-adipogenic and lipogenic transcriptome profile of SCAT, we observed an increased proportion of larger adipocytes in SCAT compared to VIAT and IMAT (Figure 5C,D). There was also an increased frequency of larger adipocytes in SCAT compared to IMAT and VIAT, while small adipocytes were more frequent in IMAT and VIAT compared to SCAT (Figure 5C,D). These results were observed through qualitative analysis of intracellular lipid accumulation in in vitro differentiated adipocytes by LipidTox staining (Figure 5E). The function of IMAT, VIAT, and SCAT adipocytes was assessed via insulin-stimulated glucose uptake (Figure 5F). Corroborating our transcriptome results, particularly when comparing IMAT vs. SCAT, we observed an increased insulin response in SCAT adipocytes compared to IMAT and VIAT cells, but it did not differ between IMAT and VIAT adipocytes.

## 4. Discussion

Our study elucidates the depot-specific transcriptome and adipocyte function in IMAT, SCAT, and VIAT in finished crossbred Angus steers. Our transcriptome data showed a pro-adipogenic/lipogenic profile of SCAT, particularly when compared to IMAT, with upregulation of key adipocyte regulators (e.g., *ADIPOQ*, *LEP*, *LIPE*, *DGAT2*) and activation of pathways of fatty acid metabolism and elongation and lipid biosynthesis and metabolism, as previously demonstrated [29,30]. Previous studies also reported the increased potential of SCAT to accumulate lipids compared to IMAT in both dairy and beef cattle [31] and of SCAT compared to omental [23] and perirenal VIAT, which had a greater lipolytic activity [32]. Remarkedly, the expression of *CFD*, a predictor of marbling expressed in IMAT fibro-adipogenic progenitors in beef steers [18], was decreased in IMAT vs. VIAT [32], similar to that observed in the present study. In line with these results, our in vitro analyses revealed a reduced ability of IMAT preadipocytes to proliferate compared to SCAT, despite the higher abundance of these cells in IMAT SVF compared to both SCAT and VIAT. These findings suggest that other factors may be limiting adipocyte differentiation and lipid accumulation in IMAT. For example, our in vitro and transcriptome results indicate a decreased insulin sensitivity of IMAT adipocytes compared to SCAT. Markers of insulin sensitivity and signaling, such as the scratch family transcriptional repressor 1 (*SCRT1*), a zinc finger transcriptional regulator of insulin in pancreatic cells [33], *TRARG1*, a positive regulator of GLUT4 trafficking and insulin sensitivity [34], and *ADIPOQ*, a key insulin sensitizing adipokine, were also downregulated in IMAT vs. SCAT. Insulin potently stimulates lipogenesis by increasing the uptake of glucose in adipocytes via recruitment of GLUT-4 to the plasma membrane, as well as activating lipogenic and glycolytic enzymes [35]. As muscle prioritizes the use of glucose as its primary fuel source [36], IMAT may become less responsive to insulin, so that muscle can develop. Our findings agree with previous studies in cattle, indicating SCAT is more responsive to insulin than IMAT, as there are higher insulin receptor concentrations and insulin binding affinity in SCAT compared to IMAT [37]. Similarly, in Holstein bulls with increased carcass fatness due to feeding a high-energy and protein diet, insulin receptor expression was lower in both SCAT and retroperitoneal adipose tissue, tending to decrease in muscle compared to bulls fed a low-energy and protein diet [38]. These findings indicate that increased fatness and diet-induced metabolic inflammation can disturb insulin signaling in adipose tissue and muscle, which may alter marbling accumulation.

Another factor interfering with IMAT deposition is its unique anatomical location in the perimysial space between the muscle fibers. While the negative effect of muscle ECM collagens and their cross-linking on beef tenderness is well established [39], how the ECM microenvironment impacts fat deposition in IMAT, SCAT, and VIAT in beef cattle needs further elucidation. Our transcriptome results revealed differences in the expression of ECM markers, showing a downregulation of metalloproteinases (e.g., *MMP14*) and tissue-inhibitor of metalloproteinases (e.g., *TIMP2*, *TIMP4*) in IMAT vs. SCAT and VIAT. In contrast, our targeted qPCR showed increased *TIMP2* and *LOX* expression in IMAT, mostly when compared with SCAT. *TIMP2* promotes intramuscular fat deposition in the muscle of chickens via the ECM receptor interaction signaling pathway [40], while LOX accelerates collagen fibril cross-linking associated with obesity [28] and fibrosis progression [41]. Notably, collagens and the connective tissue growth factor (*CTGF*) were increased in VIAT. In humans, increased expression of *CTGF* in adipose tissue was associated with increased fat mass and both skeletal muscle and liver insulin resistance [27]. Overall, these results may point to distinct mechanisms of limited fat accumulation within muscle, which seem to depend on the interactions between cells and the ECM. In line with this, previous studies have demonstrated that ECM cues influence stem cell differentiation [42]. For instance, while softer ECM substrates encouraged stem cells to favor adipogenesis, stiffer substrates favored osteogenesis [43], as increased ECM stiffness failed to upregulate adipogenic markers [44]. We have recently demonstrated that the ECM microenvironment in adipose tissue modulates the adipogenic capacity of preadipocytes in dairy cows [20]. We revealed that VIAT is stiffer than SCAT, and that VIAT ECM impaired *ADIPOQ* gene expression in SCAT adipocytes, while SCAT ECM rescued *ADIPOQ* expression in VIAT adipocytes [20]. While we have demonstrated that adipose tissue stiffness and ECM modifications modulate adipogenesis and insulin responses in a depot-specific manner in human and mouse adipocytes [45,46], how muscle ECM modulates IMAT accumulation in beef cattle is not fully understood and requires further investigation.

The location of marbling within muscle may also affect how IMAT expends energy, thus interfering with lipid accumulation. For example, in our dataset, we observed increased expression of *UCP3* and *PPARGC1B* in IMAT vs. SCAT and VIAT, which are key genes in mitochondrial biogenesis and fat oxidation and in the regulation of glucose metabolism [47]. Additionally, in our study, top upregulated KEGG pathways in IMAT vs. SCAT and VIAT included Adrenergic signaling in cardiomyocytes and Thermogenesis, both containing the enrichment of genes involved in in cellular energy metabolism and oxidative regulation, such as *ATP*, *ADCY*, and *COX.* These genes contribute to the energetic and metabolic demands of adipose tissue, particularly in depots with elevated oxidative capacity, like muscle. These results and the fact that IMAT is located within muscle suggest that intercellular signaling between these two tissues is crucial for the development of IMAT [48]. Notably, Wang et al., [49] recently reported the increased co-expression of *CPT1B* and other β-oxidation genes in muscle and liver in beef cattle, further supporting an active lipid turnover that favors IMAT accumulation. These findings also suggested that marbling results not solely from local adipogenesis but also from a systemic redistribution of hepatic lipid reserves to muscle.

Our findings highlight a pro-inflammatory transcriptional profile of VIAT, particularly when compared to IMAT. In addition to the upregulation of genes associated with activation of the inflammatory cascade, such as complement proteins (e.g., *C3*, *CFI*), pro-inflammatory cytokines and chemokines (e.g., *TNF*, *CCL5*), and markers of activated immune cells (e.g., *CD84*, *CD52*), we also observed an activation of pathways associated with immune cell migration and infiltration, as well as the increased abundance of immune cells found through flow cytometry in VIAT vs. IMAT. Our previous study using single-nuclei RNA sequencing analysis of SCAT and VAT in dairy cows similarly highlighted the pro-inflammatory profile of omental fat and identified high expression of genes encoding complement proteins [23]. In dairy cattle, VIAT inflammation has been attributed to the recruitment of macrophages into the adipose tissue, leading to chronic inflammation, as well as reduced insulin sensitivity [50,51]. Similarly, in human and animal models, visceral adiposity is associated with local and systemic inflammation, metabolic diseases such as type 2 diabetes, and insulin resistance [52,53]. Thus, in finishing or fattening beef cattle, overweight and excessive adiposity may induce a pro-inflammatory state in VIAT and contribute to higher lipid accumulation in this depot, as it does in humans and dairy cows.

In addition to the pro-inflammatory nature of VIAT, depot-specific functional differences between IMAT and SCAT have been documented across cattle breeds and species. For instance, IMAT deposition tends to be more genetically driven and varies significantly among beef breeds, with Wagyu and Angus cattle exhibiting higher marbling potential due to greater adipogenic programming and intramuscular progenitor cell abundance compared to other breeds such as Charolais or Holstein [3,54]. Transcriptome analysis of the *longissimus dorsi* muscle from the highly marbled Japanese black cattle compared with the lower marbled breeds Nanyang and Qinchuan revealed the enrichment of pathways involving biosynthesis of unsaturated fatty acids, fatty acid elongation, and metabolism, regulation of lipolysis in adipocytes, and PPAR, AMPK, and PI3K-Akt signaling [55], suggesting specific mechanisms associated with increased IMAT accumulation. In contrast, comparative analyses of SCAT across Angus, Charolais, and composite beef cattle demonstrated that SCAT fatty acid profiles differ by breed, with gene expression patterns reflecting variation in feed efficiency and lipid metabolism [56]. Across species, depot-specific differences in adipose tissue metabolism are conserved. As in our findings in cattle, in rodent models, SCAT exhibits higher insulin sensitivity and lipogenic activity, supporting efficient lipid storage and systemic metabolic health. In contrast, IMAT shows limited expandability and reduced insulin responsiveness, reflecting a more constrained metabolic profile during energy imbalance [57]. Similar patterns are observed in humans, where elevated IMAT has been associated with impaired glucose metabolism and insulin resistance [58,59].

While our combined tissue transcriptome analysis and in vitro cellular assays provided an overview of genes, pathways, and cellular mechanisms underlying the depot-specific function of IMAT, VIAT, and SCAT in beef cattle, our exploratory study also had limitations including a small sample size, the unknown specific age and background of animals, and the collection of adipose tissue at a single developmental time point, which altogether precluded the analysis of temporal and developmental context, thus restricting the interpretation of dynamic or age-dependent regulatory mechanisms of fat deposition. Additionally, the limited tissue samples from IMAT prevented the assessment of protein marker validation. Further research elucidating the relationship between transcriptional and functional differences between the distinct adipose tissue depots will be valuable in achieving greater IMAT accumulation and reduced overall economic losses associated with excessive SCAT and VIAT accumulation.

## 5. Conclusions

Our findings highlight depot-specific transcriptomic and adipocyte function between IMAT, SCAT, and VIAT. Our results demonstrate an increased propensity of SCAT to accumulate lipids, as shown by increased adipogenic and lipogenic gene expression profiles, increased adipocyte size, and insulin responses compared to both IMAT and VIAT. Our data suggest that although the increased abundance of preadipocytes in IMAT, their proliferative function and lipid accumulation are limited when compared to SCAT and VIAT adipocytes. Our results suggest that reduced adipocyte response to insulin and the muscle ECM microenvironment may contribute to limiting marbling accumulation in beef animals. The increased abundance of immune cells and upregulation of genes associated with inflammatory and immune responses in VIAT suggest its similarity with increased visceral adiposity in humans and may be associated with increased visceral fatness in finished beef steers. Identification of target genes and their depot-specific functions may allow for future selection of animals to improve carcass quality while increasing dressing percentage and yield grade.

## Figures and Tables

**Table 1:** Gene Expression TaqMan assays used in qPCR.

Gene Symbols	TaqMan Gene Expression Assay	NCBI RefSeq	Amplicon Length (Base Pairs)	Exon Boundary **
*ADIPOQ*	Bt0329341_s1	NM_174742.2	121	3
*LIPE*	Bt0325697_m1	NM_001080220.1	62	8–9
*DGAT1*	Bt03251718_g1	NM_174693.2	63	16–17
*PPARG*	Bt03217547_m1	NM_181024.2	85	6–7
*LEP*	Bt03211909_m1	NM_173928.2	88	2–3
*FASN*	Bt03210481_m1	NM_001012669.1	57	37–38
*IL6*	Bt03211905_m1	NM_173923.2	115	4–5
*TNF*	Bt03259156_m1	NM_173966.3	69	3–4
*TGFB1*	Bt04259484_m1	NM_001166068.1	60	1–2
*FN1*	Bt00415008_m1	NM_001163778.1	56	43–44
*LOX*	Bt03259128_m1	NM_173932.4	72	2–3
*TIMP2*	Bt03231007_m1	NM_174472.4	88	4–5
*EIF3K* *	Bt03226565_m1	NM_001034489.2	57	1–2
*RPS9* *	Bt03272016_m1	NM_001101152.2	62	3–4

* Reference genes used for gene expression normalization. ** Refers to the junction between two exons where the TaqMan probe and/or primers are designed.

**Table 2:** Top 10 most significant DEGs * in IMAT vs. SCAT.

Upregulated Genes IMAT vs. SCAT			
Gene Symbol	Gene Description	Log_2_Fold Change	*p*-Adjusted
*TNNC2*	Troponin C2	14.42	1.35 × 10^−68^
*CKM*	Creatine Kinase, M-Type	14.91	8.44 × 10^−52^
*ACTA1*	Actin alpha 1	16.26	1.60 × 10^−47^
*ATP2A1*	ATPase sarcoplasmic endoplasmic reticulum Ca2+ transporting 1	12.65	2.43 × 10^−44^
*ACTN3*	Actinin alpha 3	11.58	2.90 × 10^−44^
*MYLPF*	Myosin Light Chain	19.75	2.79 × 10^−43^
*TTN*	Titin	13.09	4.60 × 10^−39^
*PYGM*	Glycogen Phosphorylase	11.99	8.06 × 10^−39^
*MB*	Myoglobin	12.25	1.80 × 10^−35^
*MYL1*	Myosin Light Chain 1	19.51	2.75 × 10^−35^
**Downregulated IMAT vs. SCAT**			
**Gene Symbol**	**Gene Description**	**Log_2_Fold Change**	***p*-Adjusted**
*SCRT1*	Scratch Family Transcriptional Repressor 1	−19.84	3.18 × 10^−13^
*KRT38*	Keratin 38	−18.76	1.03 × 10^−10^
*MLC1*	Modulator Of VRAC Current 1	−7.56	2.49 × 10^−7^
*ADTRP*	Androgen-Dependent TFPI Regulating Protein	−3.39	2.83 × 10^−7^
*LASP1*	LIM And SH3 Protein 1	−1.71	1.41 × 10^−6^
*LOC100849237*	SNU13 Homolog, Small Nuclear Ribonucleoprotein pseudogene	−4.34	7.84 × 10^−7^
*SLC25A35*	Solute Carrier Family 25 Member 35	−3.72	9.63 × 10^−7^
*BAIAP2L1*	BAR/IMD Domain Containing Adaptor Protein 2 Like 1	−3.24	1.03 × 10^−6^
*XPNPEP2*	X-Prolyl Aminopeptidase 2	−4.43	1.34 × 10^−6^
*SYN2*	Synapsin II	−2.92	1.36 × 10^−6^

* DEGs were filtered by combining the highest (upregulated)/lowest (downregulated) log_2_ fold change and *p*-adjusted (Supplementary Table S3).

**Table 3:** Top 10 most significant DEGs * in VIAT vs. IMAT.

	Upregulated VIAT vs. IMAT		
Gene Symbol	Gene Description	Log_2_Fold Change	*p*-Adjusted
*SCRT1*	Scratch Family Transcriptional Repressor 1	21.13	1.65 × 10^−14^
*KRT38*	Keratin 38	19.481	1.34 × 10^−11^
*LOC101906766*	Ig lambda chain V-II region BUR-like	24.79	1.54 × 10^−9^
*NKX3−2*	NK3 Homeobox 2	10.38	3.10 × 10^−9^
*CRYBG1*	Crystallin Beta-Gamma Domain Containing 1	2.19	2.66 × 10^−6^
*PRKAR2B*	Protein Kinase CAMP-Dependent Type II Regulatory Subunit Beta	2.38	2.79 × 10^−6^
*ABO*	Alpha 1-3-N-Acetylgalactosaminyltransferase And Alpha 1-3-Galactosyltransferase	6.64	2.90 × 10^−6^
*SYN2*	Synapsin II	2.86	3.27 × 10^−6^
*TDRKH*	Tudor And KH Domain Containing	5.42	3.29 × 10^−6^
*LOC615733*	cDNA ORF clone	5.54	5.78 × 10^−6^
**Downregulated VIAT vs. IMAT**			
**Gene Symbol**	**Gene Description**	**Log_2_Fold Change**	***p*-Adjusted**
*CKM*	Creatine Kinase, M-Type	−15.19	1.58 × 10^−56^
*TNNC2*	Troponin C2	−20.29	2.31 × 10^−53^
*MB*	Myoglobin	−14.59	9.79 × 10^−52^
*ACTA1*	Actin alpha 1	−22.39	2.04 × 10^−50^
*ACTN3*	Actinin alpha 3	−11.41	1.03 × 10^−44^
*PYGM*	Glycogen Phosphorylase, muscle-associated	−12.52	3.24 × 10^−44^
*MYLPF*	Myosin Light Chain, Phosphorylatable	−19.14	1.21 × 10^−43^
*ATP2A1*	ATPase sarcoplasmic/endoplasmic reticulum Ca2+ transporting 1	−11.22	1.00 × 10^−36^
*TTN*	Titin	−11.79	2.38 × 10^−33^
*CASQ1*	Calsequestrin 2	−9.73	4.62 × 10^−33^

* DEGs were filtered by combining the highest (upregulated)/lowest (downregulated) log_2_ fold change and *p*-adjusted (Supplementary Table S3).

**Table 4:** Top 10 most significant DEGs * in VIAT vs. SCAT.

Upregulated Genes VIAT vs. SCAT			
Gene Symbol	Gene Description	Log_2_Fold Change	*p*-Adjusted
*CDH16*	Cadherin 16	20.12	3.71 × 10^−10^
*UCP3*	Uncoupling Protein 3	20.01	2.21 × 10^−7^
*FRAS1*	Fraser Extracellular Matrix Complex Subunit 1	21.02	1.11 × 10^−6^
*DSG2*	Desmoglein 2	20.48	2.86 × 10^−6^
*DSP*	Desmoplakin	7.39	2.86 × 10^−6^
*LOC505033*	Cystatin 9 Like	10.16	3.88 × 10^−5^
*SLIT2*	Slit Guidance Ligand 2	4.68	3.88 × 10^−5^
*MYBPC1*	Myosin Binding Protein C1	17.25	3.98 × 10^−5^
*MYH8*	Myosin Heavy Chain 8	20.02	5.63 × 10^−5^
*NKX3-2*	NK3 Homeobox 2	6.87	5.63 × 10^−5^
**Downregulated VIAT vs. SCAT**			
**Gene Symbol**	**Gene Description**	**Log_2_Fold Change**	***p*-Adjusted**
*HOXC10*	Homeobox C10	−9.54	1.06 × 10^−9^
*EN1*	Engrailed Homeobox 1	−7.39	4.68 × 10^−8^
*ZIC1*	Zic Family Member 1	−9.43	2.05 × 10^−7^
*PAX3*	Paired Box 3	−21.44	2.21 × 10^−7^
*HOXD8*	Homeobox D8	−3.21	2.21 × 10^−7^
*INSC*	INSC Spindle Orientation Adaptor	−8.31	7.84 × 10^−7^
*ZIC4*	Zic Family Member 4	−8.81	3.05 × 10^−5^
*ANGPTL5*	Angiopoietin Like 5	−9.49	1.99 × 10^−4^
*HOXC9*	Homeobox C9	−4.8	7.60 × 10^−5^
*MAB21L1*	Mab-21 Like 1	−5.73	3.53 × 10^−4^

* DEGs were filtered by combining the highest (upregulated)/lowest (downregulated) log_2_ fold change and *p*-adjusted (Supplementary Table S3).

## Data Availability

The transcriptome datasets generated and/or analyzed during the current study are available in the GEO repository, GSE250296 (https://www.ncbi.nlm.nih.gov/geo/query/acc.cgi?acc=GSE250296, accessed on 6 July 2025).

## Supplementary Materials

The following supporting information can be downloaded at https://www.mdpi.com/article/10.3390/biology14070848/s1. Table S1: Samples RNA integrity number (RIN) assessed by the RNA Nano 6000 Assay Kit of the Bioanalyzer 2100 system (Agilent Technologies, Santa Clara, CA, USA). Figure S1: Principal component analysis of intramuscular (IMAT), subcutaneous (SCAT), and visceral (VIAT) adipose tissue samples. Table S2: Comparison of top up- and downregulated DEGs with the inclusion and removal of the Beef 6 VIAT sample. Table S3: List of differentially expressed genes (DEGs) between IMAT and SCAT, VIAT and IMAT, and VIAT and SCAT expressed as log_2_ fold change. Table S4: List of significant KEGG pathways between IMAT and SCAT, VIAT and IMAT, and VIAT and SCAT. Table S5: List of significant GO terms between IMAT and SCAT, VIAT and IMAT, and VIAT and SCAT.

## Abbreviations

The following abbreviations are used in this manuscript:

AT	Adipose Tissue
IMAT	Intramuscular adipose tissue; marbling
SCAT	Subcutaneous adipose tissue; backfat
VIAT	Visceral adipose tissue
FFA	Free Fatty Acids
LD	Longissimus Dorsi
KRB	Krebs Ringer Buffer
SVF	Stromal Vascular Fraction
PBS	Phosphate Buffer Solution
BSA	Bovine Serum Albumin
DMEM	Dulbecco’s Modified Eagle’s Medium
IBMX	Isobutyl-1-methylaxanthine
DEG	Differentially Expressed Genes
PCA	Principal Component Analysis
KEGG	Kyoto Encyclopedia of Genes and Genomes
GO	Gene Ontology
PCR	Polymerase Chain Reaction
CT	Cycle Threshold
PFA	Paraformaldehyde
RFU	Relative fluorescent units
DAPI	diamidino-2-phenylindole

## References

[1] Nguyen D.V., Nguyen O.C., Malau-Aduli A.E.O. (2021). Main regulatory factors of marbling level in beef cattle. Vet. Anim. Sci..

[2] Harris C.L., Wang B., Deavila J.M., Busboom J.R., Maquivar M., Parish S.M., McCann B., Nelson M.L., Du M. (2018). Vitamin A administration at birth promotes calf growth and intramuscular fat development in Angus beef cattle. J. Anim. Sci. Biotechnol..

[3] Hocquette J., Gondret F., Baéza E., Médale F., Jurie C., Pethick D. (2010). Intramuscular fat content in meat-producing animals: Development, genetic and nutritional control, and identification of putative markers. Animal.

[4] USDA (2018). United States Standards for Grades of Carcass Beef. https://www.ams.usda.gov/sites/default/files/media/CarcassBeefStandard.pdf.

[5] Wheeler T.L., Cundiff L.V., Koch R.M. (1994). Effect of marbling degree on beef palatability in *Bos taurus* and *Bos indicus* cattle. J. Anim. Sci..

[6] Cianzio D.S., Topel D.G., Whitehurst G.B., Beitz D.C., Self H.L. (1982). Adipose Tissue Growth in Cattle Representing Two Frame Sizes: Distribution among Depots. J. Anim. Sci..

[7] Irshad A., Kandeepan G., Kumar S., Ashish K., Vishnuraj M., Shukla V. (2012). Factors influencing carcass composition of livestock: A review. J. Anim. Prod. Adv..

[8] Gotoh T., Albrecht E., Teuscher F., Kawabata K., Sakashita K., Iwamoto H., Wegner J. (2009). Differences in muscle and fat accretion in Japanese Black and European cattle. Meat Sci..

[9] USDA (2024). USDA Beef Carcass Price Equivalent Index Value. USDA Market News.

[10] Gotoh T., Nishimura T., Kuchida K., Mannen H. (2018). The Japanese Wagyu beef industry: Current situation and future prospects—A review. Asian-Australas. J. Anim. Sci..

[11] Smith S.B., Gill C.A., Lunt D.K., Brooks M.A. (2009). Regulation of Fat and Fatty Acid Composition in Beef Cattle. Asian-Australas. J. Anim. Sci..

[12] Sheng X., Ni H., Liu Y., Li J., Zhang L., Guo Y. (2014). RNA-seq analysis of bovine intramuscular, subcutaneous and perirenal adipose tissues. Mol. Biol. Rep..

[13] Hudson N.J., Reverter A., Griffiths W.J., Yutuc E., Wang Y., Jeanes A., McWilliam S., Pethick D.W., Greenwood P.L. (2020). Gene expression identifies metabolic and functional differences between intramuscular and subcutaneous adipocytes in cattle. BMC Genom..

[14] Ortiz-Colón G.C., Grant A.C., Doumit M.E., Buskirk D.D. (2009). Bovine intramuscular, subcutaneous, and perirenal stromal-vascular cells express similar glucocorticoid receptor isoforms, but exhibit different adipogenic capacity. J. Anim. Sci..

[15] Chung K.Y., Kim J., Johnson B.J. (2021). All-trans retinoic acid alters the expression of adipogenic genes during the differentiation of bovine intramuscular and subcutaneous adipocytes. J. Anim. Sci. Technol..

[16] May S., Savell J., Lunt D., Wilson J., Laurenz J., Smith S. (1994). Evidence for preadipocyte proliferation during culture of subcutaneous and intramuscular adipose tissues from Angus and Wagyu crossbred steers. J. Anim. Sci..

[17] Choi S.H., Silvey D.T., Johnson B.J., Doumit M.E., Chung K.Y., Sawyer J.E., Go G.W., Smith S.B. (2014). Conjugated linoleic acid (t-10, c-12) reduces fatty acid synthesis de novo, but not expression of genes for lipid metabolism in bovine adipose tissue ex vivo. Lipids.

[18] Wang L., Gao P., Li C., Liu Q., Yao Z., Li Y., Zhang X., Sun J., Simintiras C., Welborn M. (2023). A single-cell atlas of bovine skeletal muscle reveals mechanisms regulating intramuscular adipogenesis and fibrogenesis. J. Cachexia Sarcopenia Muscle.

[19] Ford H., Liu Q., Fu X., Strieder-Barboza C. (2023). White Adipose Tissue Heterogeneity in the Single-Cell Era: From Mice and Humans to Cattle. Biology.

[20] Diez J.F.F., Tegeler A.P., Flesher C.G., Michelotti T.C., Ford H., Hoque M.N., Bhattarai B., Benitez O.J., Christopher G.F., Strieder-Barboza C. (2024). Extracellular matrix modulates depot-specific adipogenic capacity in adipose tissue of dairy cattle. J. Dairy Sci..

[21] Sparks B.B., Ford H., Michelotti T.C., Strieder-Barboza C. (2025). Adipose tissue oxylipin profile changes with subclinical ketosis and depot in postpartum dairy cows. J. Dairy Sci..

[22] Hellemans J., Mortier G., De Paepe A., Speleman F., Vandesompele J. (2007). qBase relative quantification framework and software for management and automated analysis of real-time quantitative PCR data. Genome Biol..

[23] Michelotti T.C., Kisby B.R., Flores L.S., Tegeler A.P., Fokar M., Crasto C., Menarim B.C., Loux S.C., Strieder-Barboza C. (2022). Single-nuclei analysis reveals depot-specific transcriptional heterogeneity and depot-specific cell types in adipose tissue of dairy cows. Front. Cell Dev. Biol..

[24] Vijay J., Gauthier M.-F., Biswell R.L., Louiselle D.A., Johnston J.J., Cheung W.A., Belden B., Pramatarova A., Biertho L., Gibson M.J.N.M. (2020). Single-cell analysis of human adipose tissue identifies depot and disease specific cell types. Nat. Metab..

[25] Ziemke F., Mantzoros C.S. (2010). Adiponectin in insulin resistance: Lessons from translational research. Am. J. Clin. Nutr..

[26] Dandekar A.A., Wallach B.J., Barthel A., Roth R.A. (1998). Comparison of the signaling abilities of the cytoplasmic domains of the insulin receptor and the insulin receptor-related receptor in 3T3-L1 adipocytes. Endocrinology.

[27] Yoshino J., Patterson B.W., Klein S. (2019). Adipose Tissue CTGF Expression is Associated with Adiposity and Insulin Resistance in Humans. Obesity.

[28] Pastel E., Price E., Sjöholm K., McCulloch L.J., Rittig N., Liversedge N., Knight B., Møller N., Svensson P.-A., Kos K. (2018). Lysyl oxidase and adipose tissue dysfunction. Metabolism.

[29] Ou-yang Y., Dai M.-m. (2023). Screening for genes, miRNAs and transcription factors of adipogenic differentiation and dedifferentiation of mesenchymal stem cells. J. Orthop. Surg. Res..

[30] Huang W., Guo Y., Du W., Zhang X., Li A., Miao X. (2017). Global transcriptome analysis identifies differentially expressed genes related to lipid metabolism in Wagyu and Holstein cattle. Sci. Rep..

[31] Chakrabarty K., Romans J.R. (1972). Lipogenesis in the adipose cells of the bovine (*Bos taurus*) as related to their intramuscular fat content. Comp. Biochem. Physiol..

[32] Wang S., Liu J., Zhao W., Wang G., Gao S. (2023). Selection of candidate genes for differences in fat metabolism between cattle subcutaneous and perirenal adipose tissue based on RNA-seq. Anim. Biotechnol..

[33] Chriett S., Lindqvist A., Shcherbina L., Edlund A., Abels M., Asplund O., López J.M., Ottosson-Laakso E., Hatem G., Prasad R. (2021). SCRT1 is a novel beta cell transcription factor with insulin regulatory properties. Mol. Cell. Endocrinol..

[34] Duan X., Norris D.M., Humphrey S.J., Yang P., Cooke K.C., Bultitude W.P., Parker B.L., Conway O.J., Burchfield J.G., Krycer J.R. (2022). Trafficking regulator of GLUT4-1 (TRARG1) is a GSK3 substrate. Biochem. J..

[35] Kersten S. (2001). Mechanisms of nutritional and hormonal regulation of lipogenesis. EMBO Rep..

[36] Tokach R.J., Chung K.Y., Johnson B.J. (2010). Factors Affecting Intramuscular Adipose Tissue Development in Beef Cattle. https://cabcattle.com/wp-content/uploads/Texas-Tech-white-paper.pdf.

[37] McGrattan P.D., Wylie A.R.G., Nelson J. (2000). Tissue-specific differences in insulin binding affinity and insulin receptor concentrations in skeletal muscles, adipose tissue depots and liver of cattle and sheep. Anim. Sci..

[38] Kenéz Á., Bäßler S.C., Jorge-Smeding E., Huber K. (2022). Ceramide metabolism associated with chronic dietary nutrient surplus and diminished insulin sensitivity in the liver, muscle, and adipose tissue of cattle. Front. Physiol..

[39] Purslow P.P. (2014). New developments on the role of intramuscular connective tissue in meat toughness. Annu. Rev. Food Sci. Technol..

[40] Cui H.-x., Luo N., Guo L.-p., Liu L., Xing S.-y., Zhao G.-p., Wen J. (2023). TIMP2 promotes intramuscular fat deposition by regulating the extracellular matrix in chicken. J. Integr. Agric..

[41] Unamuno X., Gómez-Ambrosi J., Becerril S., Álvarez-Cienfuegos F.J., Ramírez B., Rodríguez A., Ezquerro S., Valentí V., Moncada R., Mentxaka A. (2022). Changes in mechanical properties of adipose tissue after bariatric surgery driven by extracellular matrix remodelling and neovascularization are associated with metabolic improvements. Acta Biomater..

[42] Iwayama T., Steele C., Yao L., Dozmorov M.G., Karamichos D., Wren J.D., Olson L.E. (2015). PDGFRα signaling drives adipose tissue fibrosis by targeting progenitor cell plasticity. Genes Dev..

[43] Guvendiren M., Burdick J.A. (2012). Stiffening hydrogels to probe short-and long-term cellular responses to dynamic mechanics. Nat. Commun..

[44] Young D.A., Choi Y.S., Engler A.J., Christman K.L. (2013). Stimulation of adipogenesis of adult adipose-derived stem cells using substrates that mimic the stiffness of adipose tissue. Biomaterials.

[45] Strieder-Barboza C., Baker N.A., Flesher C.G., Karmakar M., Patel A., Lumeng C.N., O’Rourke R.W. (2020). Depot-specific adipocyte-extracellular matrix metabolic crosstalk in murine obesity. Adipocyte.

[46] Strieder-Barboza C., Baker N.A., Flesher C.G., Karmakar M., Neeley C.K., Polsinelli D., Dimick J.B., Finks J.F., Ghaferi A.A., Varban O.A. (2019). Advanced glycation end-products regulate extracellular matrix-adipocyte metabolic crosstalk in diabetes. Sci. Rep..

[47] Schrauwen P., Hesselink M. (2002). UCP2 and UCP3 in muscle controlling body metabolism. J. Exp. Biol..

[48] Roberts S., Lancaster P., DeSilva U., Horn G., Krehbiel C. (2015). Coordinated gene expression between skeletal muscle and intramuscular adipose tissue in growing beef cattle. J. Anim. Sci..

[49] Wang S., Liu T., Peng P., Fu Y., Shi S., Liang S., Chen X., Wang K., Zhou R. (2025). Integrated Transcriptomic Analysis of Liver and Muscle Tissues Reveals Candidate Genes and Pathways Regulating Intramuscular Fat Deposition in Beef Cattle. Animals.

[50] Moisá S.J., Ji P., Drackley J.K., Rodriguez-Zas S.L., Loor J.J. (2017). Transcriptional changes in mesenteric and subcutaneous adipose tissue from Holstein cows in response to plane of dietary energy. J. Anim. Sci. Biotechnol..

[51] Contreras G.A., Kabara E., Brester J., Neuder L., Kiupel M. (2015). Macrophage infiltration in the omental and subcutaneous adipose tissues of dairy cows with displaced abomasum. J. Dairy Sci..

[52] Mathis D. (2013). Immunological goings-on in visceral adipose tissue. Cell Metab..

[53] Deiuliis J.A., Oghumu S., Duggineni D., Zhong J., Rutsky J., Banerjee A., Needleman B., Mikami D., Narula V., Hazey J. (2014). CXCR3 modulates obesity-induced visceral adipose inflammation and systemic insulin resistance. Obesity.

[54] Wang Y.H., Bower N., Reverter A., Tan S., De Jager N., Wang R., McWilliam S., Cafe L., Greenwood P., Lehnert S. (2009). Gene expression patterns during intramuscular fat development in cattle. J. Anim. Sci..

[55] Yu H., Yu S., Guo J., Wang J., Mei C., Abbas Raza S.H., Cheng G., Zan L. (2024). Comprehensive Analysis of Transcriptome and Metabolome Reveals Regulatory Mechanism of Intramuscular Fat Content in Beef Cattle. J. Agric. Food Chem..

[56] Zhou M., Zhu Z., Sun H.-Z., Zhao K., Dugan M.E.R., Bruce H., Fitzsimmons C., Li C., Guan L.L. (2022). Breed dependent regulatory mechanisms of beneficial and non-beneficial fatty acid profiles in subcutaneous adipose tissue in cattle with divergent feed efficiency. Sci. Rep..

[57] Schoettl T., Fischer I.P., Ussar S. (2018). Heterogeneity of adipose tissue in development and metabolic function. J. Exp. Biol..

[58] Goodpaster B.H., He J., Watkins S., Kelley D.E. (2000). Skeletal muscle lipid content and insulin resistance: Evidence for a paradox in endurance-trained athletes. J. Clin. Endocrinol. Metab..

[59] Addison O., Marcus R.L., Lastayo P.C., Ryan A.S. (2014). Intermuscular fat: A review of the consequences and causes. Int. J. Endocrinol..

