# Supplementary figures and images for: Transcriptome and Cellular Evidence of Depot-Specific Function in Beef Cattle Intramuscular, Subcutaneous, and Visceral Adipose Tissues
Source: Biology (Basel). 2025 Jul 11;14(7):848. doi: 10.3390/biology14070848 (PMC12292588; doi:10.3390/biology14070848)

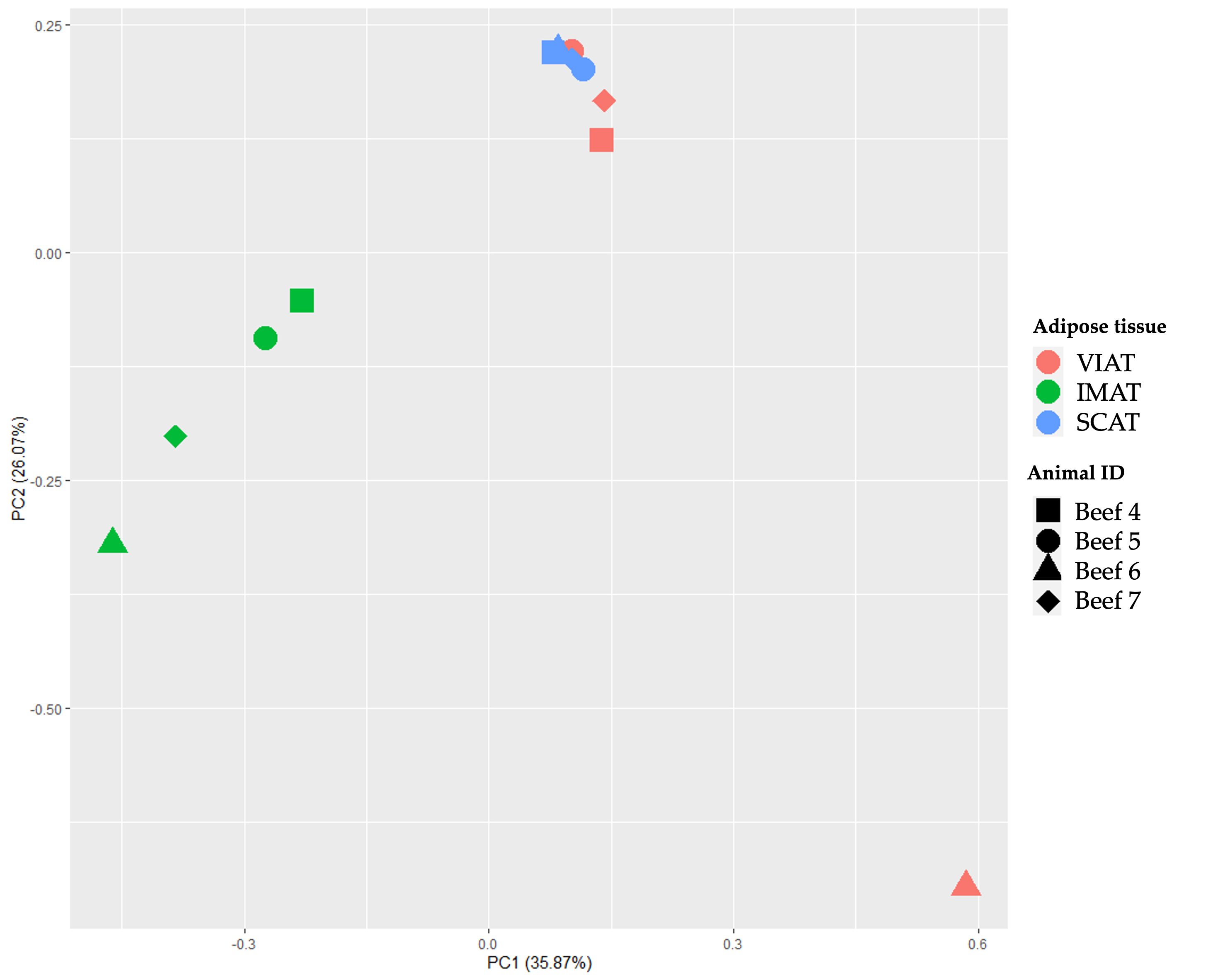

Supplement: Supplementary file 1 [file biology-14-00848-s001.zip › biology-3683260-supplementary/Supplementary materials_Tegeler et al 2025/Suppl. Figure S1.png]
